# Unique features of *Entamoeba histolytica* glycerophospholipid metabolism; has the *E*. *histolytica* lipid metabolism network evolved through gene loss and gain to enable parasitic life cycle adaptation?

**DOI:** 10.1128/msphere.00174-23

**Published:** 2023-08-16

**Authors:** Fumika Mi-ichi, Hiroshi Tsugawa, Hiroki Yoshida, Makoto Arita

**Affiliations:** 1 Central Laboratory, Institute of Tropical Medicine (NEKKEN), Nagasaki University, Nagasaki, Japan; 2 Division of Molecular and Cellular Immunoscience, Department of Biomolecular Sciences, Faculty of Medicine, Saga University, Saga, Japan; 3 Department of Biotechnology and Life Science, Tokyo University of Agriculture and Technology, Tokyo, Japan; 4 Laboratory for Metabolomics, RIKEN Center for Integrative Medical Sciences, Yokohama, Japan; 5 Graduate School of Medical Life Science, Yokohama City University, Yokohama, Japan; 6 Division of Physiological Chemistry and Metabolism, Graduate School of Pharmaceutical Sciences, Keio University, Tokyo, Japan; 7 Human Biology-Microbiome-Quantum Research Center (WPI-Bio2Q), Keio University, Tokyo, Japan; University of California, Davis, California, USA

**Keywords:** lipid metabolism, *Entamoeba*, lateral gene transfer

## Abstract

*Entamoeba histolytica*, a protozoan parasite, causes amoebiasis, which is a global public health problem. During the life cycle of this parasite, the properties of the cell membrane are changed markedly. To clarify the mechanism of membrane lipid changes, we exploited state-of-the-art untargeted lipidomic analysis, and atypical features of glycerophospholipids, lysoglycerophospholipids, and sphingolipids were observed compared with human equivalents. Here, we overview an entire *E. histolytica* glycerophospholipid metabolic pathway based on re-evaluated whole lipidome and genome along with the results of metabolic labeling experiments. We also discuss whether the *E. histolytica* lipid metabolism network, including the glycerophospholipid metabolic pathway, has unique features necessary for parasitic life cycle adaptation through gene loss and/or gain, and raise important questions involving biochemistry, molecular cell biology, and physiology underlying this network. Answering these questions will advance the understanding of *Entamoeba* physiology and will provide potential targets to develop new anti-amoebiasis drugs.

## OPINION/HYPOTHESIS

*Entamoeba histolytica* is a protozoan parasite that causes amoebiasis, a global public health problem that has inadequate clinical options as well as high morbidity and mortality rates (1). This parasite maintains its life cycle by alternating between two forms, which have drastically different membrane properties. One form is trophozoites, proliferative amoeboid cells, in which vesicular trafficking and molecule transport across the plasma membrane are highly active (2 – 4). The other form is cysts, nonmotile round dormant cells, in which the number of intracellular vesicles is decreased and the plasma membrane becomes impermeable to substances (2, 5). To clarify the mechanism of membrane lipid changes throughout the life cycle of *Entamoeba* parasites (*E. histolytica* and *Entamoeba invadens*, model for encystation), we performed state-of-the-art liquid chromatography tandem mass spectrometry-based untargeted lipidomics (5, 6). For instance, dihydroceramide (Cer-NDS) containing very long *N*-acyl chains (≥26 carbons) was selectively synthesized as the terminal product of a *de novo* metabolic pathway during encystation, and this pathway was critical in the establishment of membrane impermeability. We also showed structural uniqueness of glycerophospholipids (GPLs) and lysoglycerophospholipids (LGPLs). Many of these carried one very long (26–30 carbons) acyl chain, and some carried saturated- and short/medium (6–12 carbons) acyl chains as the acyl partner (5). Parasitic organisms have typically evolved by gene loss and/or gain (and plausibly lateral gene transfer [7]) during adaptation to their host; therefore, we assumed that this would be the case for *Entamoeba* GPL metabolism, which generates structural uniqueness among GPLs/LGPLs. To assess this, we re-evaluated the latest *E. histolytica* lipidomics (index number [IN] DM0036 in the RIKEN DROP Met; http://prime.psc.riken.jp/menta.cgi/prime/drop_index) and genomics (AmoebaDB Release 61; https://amoebadb.org/amoeba/app) data and integrated them with metabolic labeling results. We then overviewed an entire *E. histolytica* GPL metabolic pathway with reference to KEGG (https://www.genome.jp/kegg/). We also discussed whether the *Entamoeba* lipid metabolism network generates unique features necessary for adaptation of its parasitic life cycle by gene loss and/or gain.

### Unique features of GPL structures and their biosynthesis in *E*. *histolytica*; rare acyl chains and loss of genes for ubiquitous enzymes

Re-evaluation of *E. histolytica* lipidomics data (IN DM0036 in the RIKEN DROP Met) showed that major phosphatidylethanolamines (PEs), phosphatidylserines (PSs), and phosphatidylinositols (PIs) carried one very long acyl chain (C24–C30) (Fig. S1). These acyl chains are rarely found in mammals, such as human and mouse (Supplemental Data 1 in reference 6). Among these PEs, C_18:2_ was the most abundant acyl partner, followed by C_20:4_, C_18:1_, and C_18:3_ (PE in Fig. S1). Acyl partners of PSs and PIs were different from those of PEs. In almost all PSs, saturated medium-length acyl chains (C8–C10) were the partner, whereas in PIs, saturated long chains (C12–C16) were the major partner (PS and PI in Fig. S1). Of note, like very long chains, saturated C8–C10 acyl chains are not the major products in mammals (Supplemental Data 1 in reference 6). In contrast to the above three GPLs, phosphatidylcholines (PCs) that carry very long acyl chains were at far lower levels, and major PCs carried very similar acyl combinations to those in mammals; for instance, C_16:0_ /C_18:1 or 18:2_, C_18:0_ /C_18:2_, and C_18:1 or 18:2_ /C_18:2_ (PC in Fig. S1). In parallel with the acyl chain profiles of GPLs, very long acyl chains were present in LPEs, LPSs, and LPIs, while LPCs with very long acyl chains were not present (LPE, LPS, LPI, and LPE in Fig. S1).

**Fig 1:**
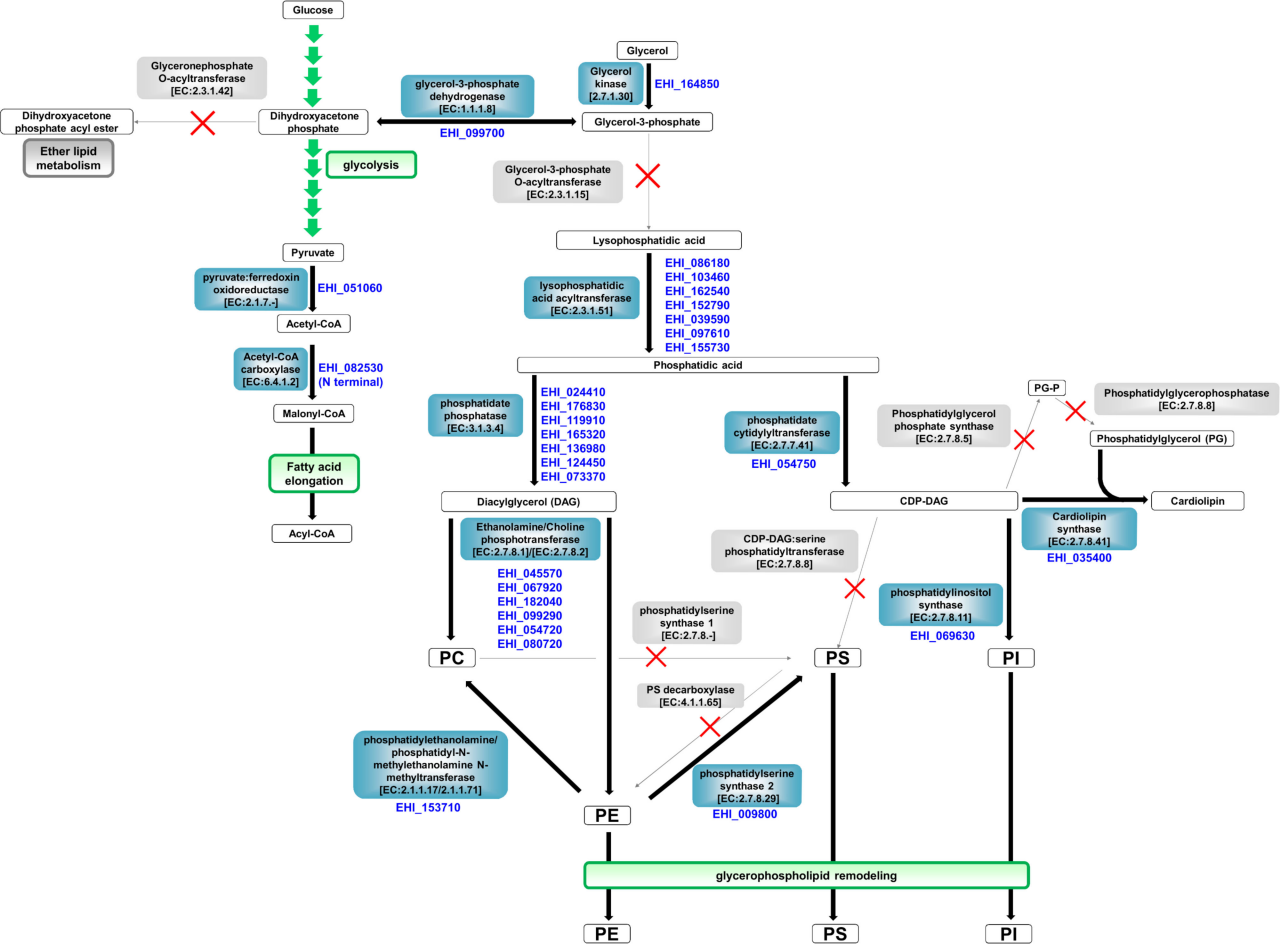
Deduced *E. histolytica* glycerophospholipid (GPL) metabolic pathway. *E. histolytica* gene IDs in AmoebaDB are shown. Orthologs of *E. histolytica* enzymes in its GPL metabolic pathway are encoded in the genome of *E. invadens*, model for encystation (see Fig. S2). GPAT, glycerol-3-phosphate *O*-acyltransferase; LPAAT, lysophosphatidic acid acyltransferase; LPLAT, lysophospholipid acyltransferase; PA, phosphatidic acid; PC, phosphatidylcholine; PE, phosphatidylethanolamine; PI, phosphatidylinositol; PS, phosphatidylserine.

**Fig 2:**
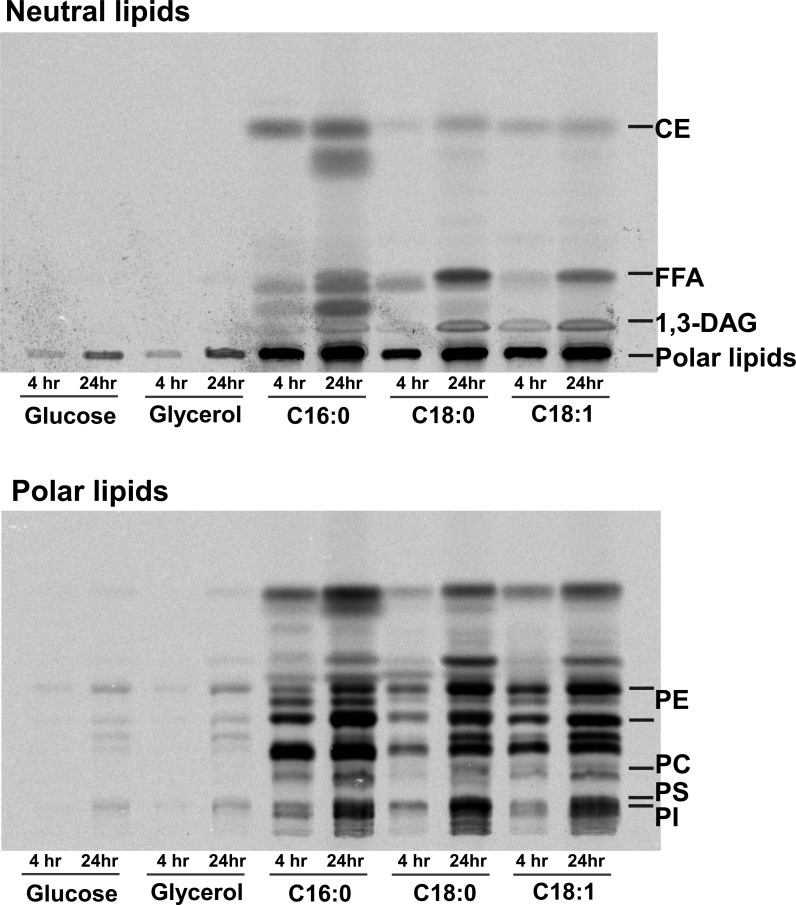
Metabolic labeling of *E. histolytica. E. histolytica*, which was maintained as described (9), was metabolically labeled using D-[U-^14^C]-glucose and -glycerol, and [1-^14^C]-palmitic, -stearic and -oleic acid (Perkin Elmer, Sugar Land, TX, USA) for 4 and 24 hours as described in reference 5. Radiolabeled glucose and glycerol were directly added to culture media (4 µCi/mL of medium), whereas radiolabeled fatty acids were reconstituted with 5% lipid-free bovine serum albumin (Sigma-Aldrich, St. Louis, MO, USA; catalog no. A7511) and were then added to culture media (1 µCi/mL of medium). Total lipids were extracted from radiolabeled cells, which were collected as previously described (5). The lipids extracted from 5.5 × 10^5^ cells were resolved by thin-layer chromatography (TLC) on Silica Gel 60 high-performance TLC plates (Merck, Darmstadt, Germany) using chloroform-methanol-15 N NH3 (60:35:8 [vol/vol/vol]) or N-hexane-diethyl ether-acetic acid (70:30:1 [vol/vol/vol]) for polar lipids or neutral lipids, respectively. Each spot on TLC plates was quantified using a Fuji imaging analyzer and Multi Gauge 2.2 software (FLA-7000; Fujifilm, Tokyo, Japan). CE, cholesteryl ether; DAG, diacyl glycerol; FFA, free fatty acid; PC, phosphatidylcholine; PE, phosphatidylethanolamine; PI, phosphatidylinositol; PS, phosphatidylserine.

Based on *E. histolytica* lipidome (Fig. S1) and reassessed genomic data (Supplemental text; AmoebaDB), we overviewed the entire *E. histolytica* GPL metabolic pathway (Fig. 1). A hallmark of the deduced pathway was that several genes encoding ubiquitous enzymes were lost. The most notable one was loss of the gene encoding glycerol-3-phosphate *O*-acyltransferase (GPAT), an initial enzyme of typical *de novo* GPL synthesis. Consistently, the absence of GPAT activity was shown by the metabolic labeling of *E. histolytica*. Signals corresponding to lipids from ^14^C-labeled glucose and glycerol were barely detectable at 4 hours and were at low levels at 24 hours. In contrast, ^14^C-palmitic (C_16:0_), -stearic (C_18:0_), and -oleic (C_18:1_) acids were clearly incorporated into a variety of lipids, such as PC, PE, PI, and PS, even by short-time labeling (Fig. 2). Faint lipid bands detected at 24 hours by ^14^C-glucose and -glycerol labeling were probably produced via pyruvate, the terminal product of glycolysis, which can be converted to malonyl-coenzyme A (CoA), and then incorporated into lipids by fatty acid elongation (Polar lipids in Fig. 2; see Fig. 1). Fatty acyl-CoAs synthesized via this pathway are also possible to be incorporated into neutral lipids, but the signals could not be detected at the present experiment condition. Metabolic labeling using ^14^C-pyruvate will be a confirmatory experiment. Importantly, the metabolic labeling results are consistent with a previous finding that consumed glucose is mainly catabolized into gases and volatile liquids in *E. histolytica* (>80% of the total carbon/consumed glucose) (8).

**Fig 3:**
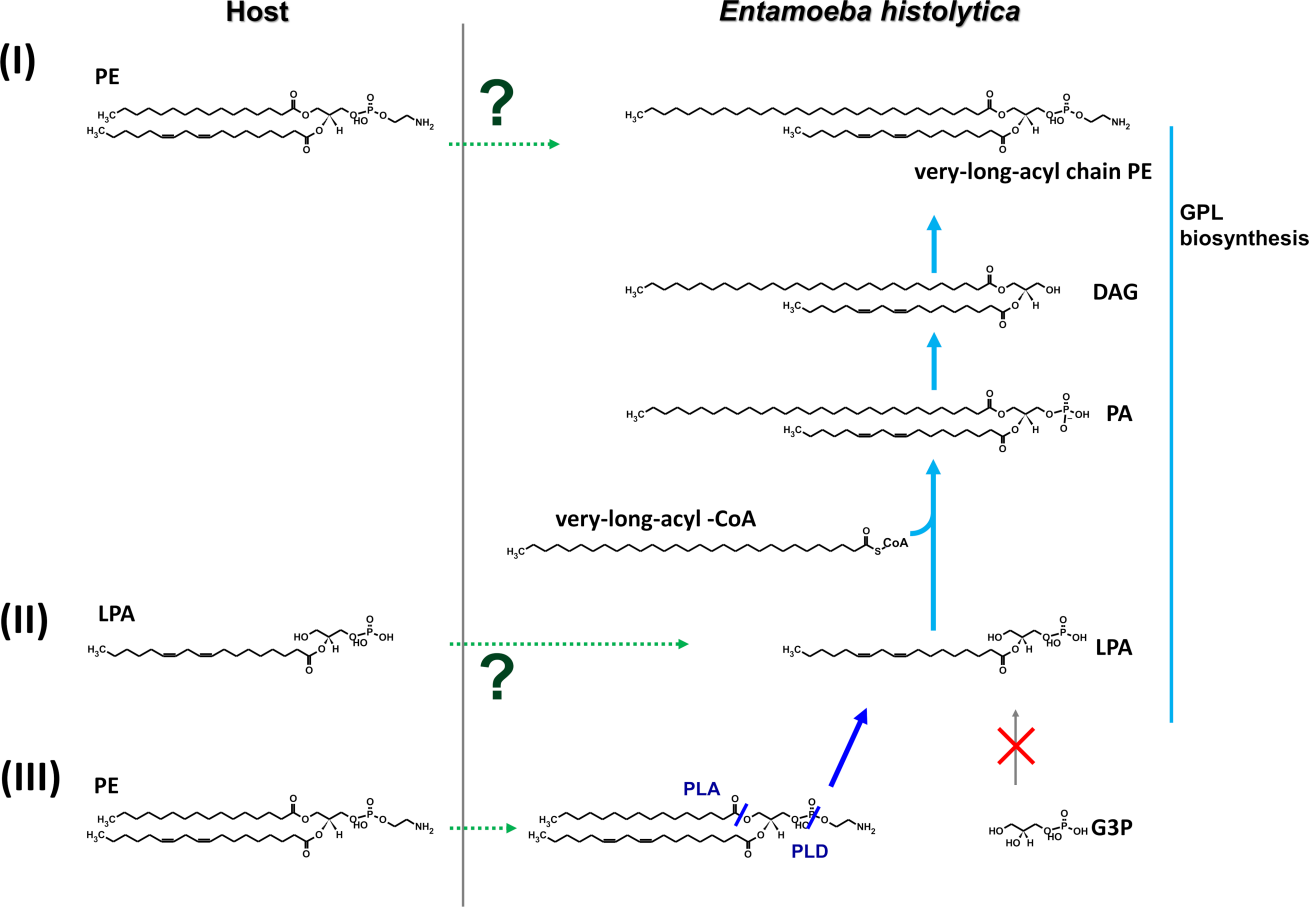
Schematic illustration for potential routes to bypass GPAT-mediated step in *E. histolytica* GPL biosynthesis. Three potential routes (I to III) are shown for providing a PE as the example that enable bypass the GPAT-mediated step. In routes I and II, *E. histolytica* scavenges a final product, PE, and a precursor, LPA, respectively, from the host, and then uses as they are. In route III, *E. histolytica* scavenges PE and then degrades it by PLA and PLD to provide LPA for its GPL biosynthesis. CoA, coenzyme A; DAG, diacylglycerol; G3P, glycerol-3-phosphate; GPAT, glycerol-3-phosphate *O*-acyltransferase; GPL, glycerophospholipid; LPA, lysophosphatidic acid; PA, phosphatidic acid; PE, phosphatidylethanolamine; PLA, phospholipase A; PLD, phospholipase D.

An important question to be answered, therefore, is how does *E. histolytica* provide diacyl-GPLs, such as PC, PE, PI, and PS, without GPAT. Several answers are possible (Fig. 3). (I) *E. histolytica* scavenges host-derived diacyl-GPLs and utilizes them as they are. (II) *E. histolytica* salvages host-derived lysophosphatidic acid (LPA), a GPAT-mediated reaction product, for its GPL biosynthesis. (III) *E. histolytica* scavenges host-derived diacyl-GPLs and degrades them to provide LPA for its GPL biosynthesis. We consider the third possibility (III) to be the most likely because of the following: the different acyl chain profiles of *E. histolytica* GPLs compared with those of host GPLs (Fig. S1), low availability of LPA in the host because LPA production is regulated at multiple levels according to its requirement (10), and the presence of downstream enzymes for *E. histolytica* GPL biosynthesis (Fig. 1).

To provide LPA, which is an initial substrate for *de novo* diacyl-GPL synthesis in *E. histolytica*, we assumed the presence of host-diacyl-GPL scavenging machinery and a set of two phospholipases (III in Fig. 3). This putative set consists of phospholipase A_1_ or A_2_ (PLA_1_ or A_2_) and phospholipase D (PLD). Putative PLA and PLD are both encoded in *E. histolytica* genome (AmoebaDB; PLA, EHI_073330, EHI_167000, EHI_200740, EHI_014360, EHI_050610, EHI_065690, EHI_068060, EHI_153390, EHI_103880, and EHI_105850; PLD, EHI_082560, and EHI_146550). In AmoebaDB, the 10 genes encoding putative PLA are annotated to encode proteins that contain a patatin-like domain. Patatin is a potato tuber storage protein, and related proteins in animals, bacteria, and plants are characterized by having a catalytic center that consists of several specific sequence elements (11). Importantly, some of these show PLA activity (11) and PLA activities in *E. histolytica* have been shown (12). Therefore, these lines of evidence and also the most reasonable explanation for unique acyl chain profiles of *E. histolytica* GPLs (see below) indicate that experimental validation of the presence of host-diacyl-GPL scavenging machinery is warranted (Fig. 3).

In *de novo* PC/PE synthesis, all enzymes involved in the “Kennedy pathway” are encoded in *E. histolytica* genome (13) (AmoebaDB). Consistently, the acyl chain profile of diacylglycerols (DAGs), the immediate precursor for PC/PE in the pathway, was very similar to that of PEs (PE and DAG in Fig. S1). Meanwhile, PCs carrying very long acyl chains were also detected, but at a very low level (PC in Fig. S1). The observed differences in the acyl chain profiles between PEs and PCs are likely to reflect the substrate preference of PC and PE synthases; PC synthase may preferentially use DAG carrying mammalian-type di-acyl chains as a substrate, whereas PE synthase may use available DAG as a substrate. Incidentally, PE *N*-methyltransferase, which catalyzes polar head group conversion of PE to PC, is encoded in *E. histolytica* genome, but the acyl chain profile of PC was not similar to that of PEs (PE and PC in Fig. S1). PS decarboxylase, which catalyzes polar head group conversion of PS to PE, is not encoded in *E. histolytica* genome (AmoebaDB), indicating that the Kennedy pathway is the major route for *de novo* PC/PE synthesis in *E. histolytica*.

In *de novo* PS synthesis, the genes encoding CDP-DAG:serine phosphatidyltransferase and PS synthase 1, which catalyze the transfer of a CDP-DAG PA moiety to serine and the exchange of a PC choline moiety with serine, respectively, are absent from *E. histolytica* genome (Fig. 1). These enzymes are responsible for two out of three routes for PS synthesis in mammals. *De novo* PS synthesis in *E. histolytica* would therefore uniquely rely on the third route, in which PS synthase 2 catalyzes the exchange of a PE ethanolamine moiety with serine. Although overall acyl chain profile similarity between PEs and PSs was not very high, both lipid classes that carried very long and saturated medium-length acyl chains were found (PE and PS in Fig. S1). This profile may indicate the preference of PS synthase 2 for PE with very long and saturated medium-length acyl chains as substrates.

Gene loss is also seen in *de novo* PG synthesis (Fig. 1). Two genes encoding enzymes that are responsible for the sequential reactions to produce PGs from CDP-DAG are absent from *E. histolytica* genome (Fig. 1). Although cardiolipins (CLs) and PGs were not detected (IN DM0036 in the RIKEN DROP Met), a protein showing significant homology to CL synthase is encoded in *E. histolytica* genome (13, 14) and was previously shown to be a mitosomal protein by organelle proteomic analysis (EHI_035400 in AmoebaDB; XP_656112 in reference 15). The role of this protein in *E. histolytica* therefore warrants future investigation.

The gene encoding glyceronephosphate *O*-acyl transferase, the other initial enzyme in typical *de novo* GPL synthesis, is also lost, which is consistent with the absence of both alkyl- and alkenyl-GPLs in *E. histolytica*.

Should the above scenario for *E. histolytica* GPL metabolism be correct, the unique feature of PEs, PIs, and PSs carrying saturated C6–C12 acyl chains together with very long chains (C24–C30) needs to be explained. Remodeling of host diacyl-GPL-derived acyl chains linked to these three lipid classes with a saturated chain is the most plausible explanation. Consistently, at least seven LPA acyltransferase (LPAAT) isozymes are encoded in the *E. histolytica* genome (AmoebaDB) and some of them may have acyl-CoA:lysophospholipid acyltransferase (LPLAT) activity, as has been shown for mammalian homologs (16). PS synthase 2 may also be involved in providing PS carrying the above acyl combinations from the corresponding PE.

Meanwhile, the supply of very-long-chain (C24–C30) and saturated medium-length/long-chain (C8–C15) fatty acyl-CoAs, the reactive form of fatty acid in GPL metabolism, also needs to be considered. As a source of fatty acids, *E. histolytica* is thought to mainly rely on scavenging from the external milieu. This is because neither type I nor type II fatty acid synthases, which are responsible for *de novo* fatty acid synthesis, are genomically encoded (17). The above fatty acids are, however, rare or at low levels in the host (Supplemental Data 1 in reference 6). We, therefore, assume that the *E. histolytica* elongation system, which consists of four cyclic reactions that add two acyl units to a substrate acyl-CoA at every cycle (5, 9), is responsible for providing very-long-chain acyl-CoAs. Of note, *E. histolytica* elongase genes, which encode the first enzyme in this elongation cycle, are closely related to green algae and plant counterparts (17), indicating that *E. histolytica* acquired these genes by lateral gene transfer. Furthermore, the *E. histolytica* fatty acid elongation system is also potentially responsible for providing saturated medium-length/long-chain acyl-CoA. *E. histolytica* cannot shorten scavenged fatty acids because fatty acid β-oxidation enzymes, which degrade fatty acids by releasing two acyl units at every cycle, are not genomically encoded (17) (AmoebaDB). Therefore, in *E. histolytica*, an elongase-mediated *de novo* pathway is likely to be responsible for providing fatty acyl-CoAs. This is the case in *Trypanosoma brucei*, which does synthesize a variety of fatty acids but whose genome encodes neither type I nor type II fatty acid synthases (like *E. histolytica*) (18). It is worth mentioning that fatty acid desaturases are also not encoded in *E. histolytica* genome (AmoebaDB). Therefore, *E. histolytica* fatty acid elongation plays crucial roles in GPL metabolism by providing a broad range of required fatty acyl-CoAs.

### Conclusions and future perspectives

Here, we overviewed the entire *E. histolytica* GPL metabolic pathway and described its close linkage with fatty acid metabolism and also its unique features. It is worth mentioning that GPL and fatty acid metabolism in *E. histolytica* exist in a network with sulfolipid metabolism, which is important in establishing the parasitic life cycle (9, 15, 19 – 21). Furthermore, in *E. histolytica* sulfolipid metabolism, genes encoding sulfate activation enzymes and sulfotransferases may have been transferred from other organisms (9, 15, 21). Gene losses and gains usually have causal connections with parasite adaptation to the host. We, therefore, assume that atypical features of *E. histolytica* GPL metabolism result from gene losses and/or gains during the course of its host adaptation and that further biochemical investigation of *E. histolytica* GPL metabolism is warranted.

This study also raises important questions. How is the *E. histolytica* lipid metabolism network, which consists of fatty acid, sulfolipid, sphingolipid, and GPL metabolism (this study; 5, 9, 21), regulated? What is the molecular mechanism underlying this regulation? What are the biological roles of unique acyl chains linked to various lipids? What are the biological consequences that result from the interactions among the unique lipid species, and what are the underlying mechanisms? Answering these questions will deepen our understanding of how membrane properties are controlled in *E. histolytica*. This ultimate goal will be achieved using biochemical and molecular cell approaches and new interdisciplinary approaches from chemistry and biophysics. Such studies will advance the understanding of *Entamoeba* biology, and will provide potential targets to develop new drugs against amoebiasis.

## SUPPLEMENTAL MATERIAL

Fig. S1 msphere.00174-23-s0001.tif Lipid profiles in *E. histolytica*.

Fig. S2 msphere.00174-23-s0002.tif Deduced *E. invadens* glycerophospholipid (GPL) metabolic pathway.

Supplemental material msphere.00174-23-s0003.docx Supplemental text and figure legends.

ASM does not own the copyrights to Supplemental Material that may be linked to, or accessed through, an article. The authors have granted ASM a non-exclusive, world-wide license to publish the Supplemental Material files. Please contact the corresponding author directly for reuse.
