# Supplementary figures and images for: Unique features of Entamoeba histolytica glycerophospholipid metabolism; has the E. histolytica lipid metabolism network evolved through gene loss and gain to enable parasitic life cycle adaptation?
Source: mSphere. 2023 Aug 16;8(5):e00174-23. doi: 10.1128/msphere.00174-23 (PMC10597341; doi:10.1128/msphere.00174-23)

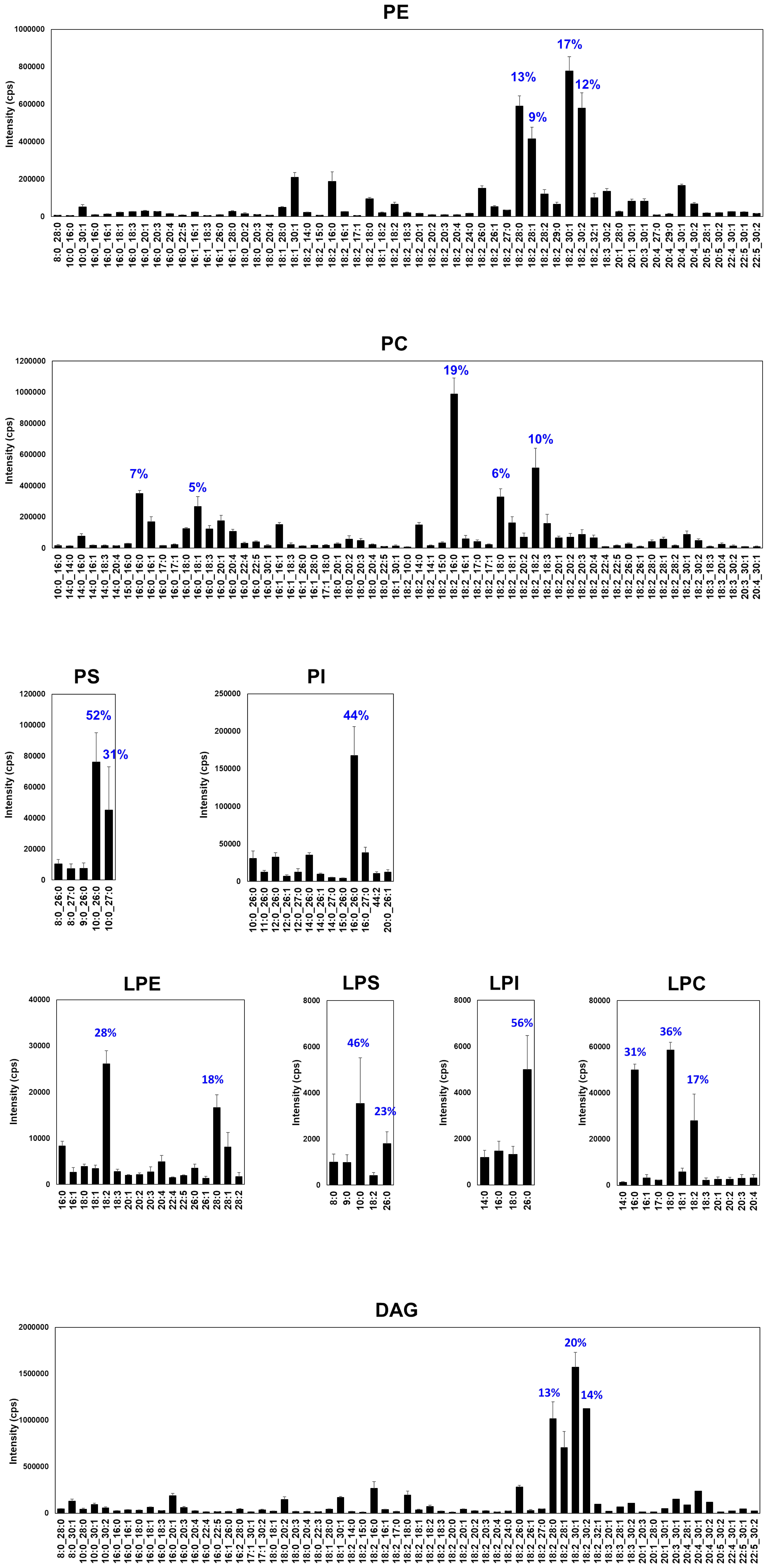

Supplement: Fig. S1 — Lipid profiles in E. histolytica. [file msphere.00174-23-s0001.tif]

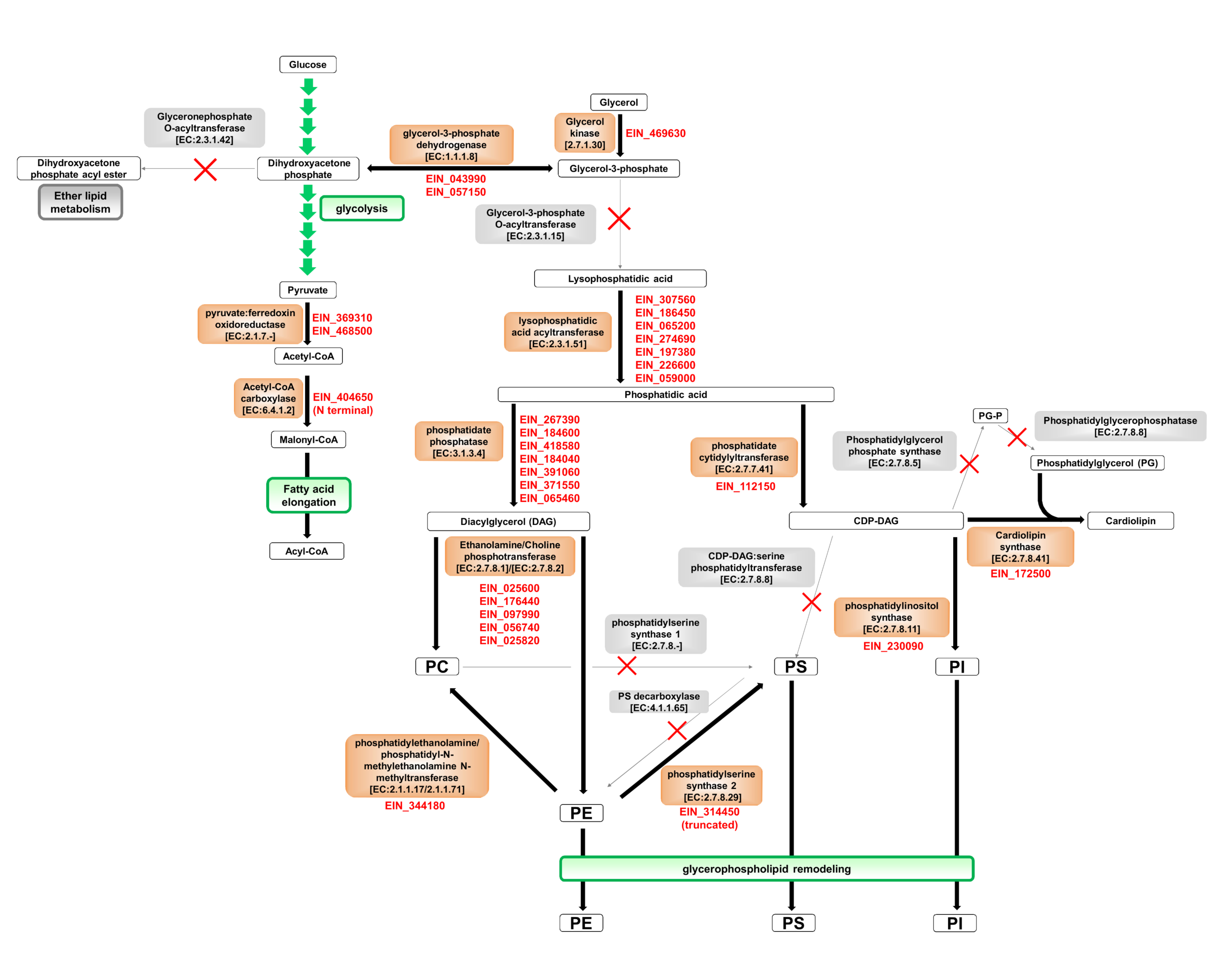

Supplement: Fig. S2 — Deduced E. invadens glycerophospholipid (GPL) metabolic pathway. [file msphere.00174-23-s0002.tif]
