# Supplementary material for: Unique features of Entamoeba histolytica glycerophospholipid metabolism; has the E. histolytica lipid metabolism network evolved through gene loss and gain to enable parasitic life cycle adaptation?
Source: mSphere. 2023 Aug 16;8(5):e00174-23. doi: 10.1128/msphere.00174-23 (PMC10597341; doi:10.1128/msphere.00174-23)
Supplement: Supplemental material — Supplemental text and figure legends. [file msphere.00174-23-s0003.docx]

**Supplemental Materials**

**Supplemental text**

**Methods**

*Re-evaluation of* *E*. *histolytica lipidomic data*

Raw data for control strain data (n=3) in IN DM0036 in the RIKEN DROP Met were analyzed and are shown as the mean ± standard deviation.

*Re-evaluation of E*. *histolytica and E*. *invadens genomic data in AmoebaDB*

The genes listed in KEGG were searched for again in AmoebaDB and occasionally used in NCBI BLAST searches while the genes not listed in KEGG were identified by NCBI BLAST searches using related genes in *Dictyostelium*, *Acanthamoeba*, yeast, plants, and mammals.

**Fig. S1. Lipid profiles in *E. histolytica*.**

LC-MS/MS signal intensity levels are shown. Relative value of signal intensity of each lipid species is calculated to total amount of all detectable species in each lipid class as 100% and the values of major species in each class are shown.

**Fig S2. Deduced *E*. *invadens* glycerophospholipid (GPL) metabolic pathway.**

*E*. *invadens* gene IDs in AmoebaDB are shown. GPAT, glycerol-3-phosphate *O*-acyltransferase; LPAAT, lysophosphatidic acid acyltransferase; LPLAT; lysophospholipid acyltransferase; PA, phosphatidic acid; PC, phosphatidylcholine; PE, phosphatidylethanolamine; PI, phosphatidylinositol; PS, phosphatidylserine.
